# DOCSIC: A Mean-Field Method for Orbital-by-Orbital Self-Interaction Correction

**DOI:** 10.1021/acs.jpca.4c02801

**Published:** 2024-07-10

**Authors:** Juan E. Peralta, Veronica Barone, Juan I. Melo, Diego R. Alcoba, Gustavo E. Massaccesi, Luis Lain, Alicia Torre, Ofelia B. Oña

**Affiliations:** † Department of Physics, Central Michigan University, Mount Pleasant, Michigan 48859, United States; ‡ Universidad de Buenos Aires, Facultad de Ciencias Exactas y Naturales, Departamento de Física. Ciudad Universitaria, 1428 Buenos Aires, Argentina; § CONICET - Universidad de Buenos Aires, Instituto de Física de Buenos Aires (IFIBA), Ciudad Universitaria, 1428 Buenos Aires, Argentina; ∥ Departamento de Ciencias Exactas, Ciclo Básico Común, Universidad de Buenos Aires, Ciudad Universitaria, 1428 Buenos Aires, Argentina; ⊥ Instituto de Investigaciones Matemáticas “Luis A. Santaló” (IMAS), Consejo Nacional de Investigaciones Científicas y Técnicas, Universidad de Buenos Aires, Ciudad Universitaria, 1428 Buenos Aires, Argentina; # Departamento de Química Física, Facultad de Ciencia y Tecnología, Universidad del País Vasco, Apdo. 644, E-48080 Bilbao, Spain; ○ Instituto de Investigaciones Fisicoquímicas Teóricas y Aplicadas, Universidad Nacional de La Plata, Consejo Nacional de Investigaciones Científicas y Técnicas, Diag. 113 y 64 (S/N), Sucursal 4, CC 16, 1900 La Plata, Argentina

## Abstract

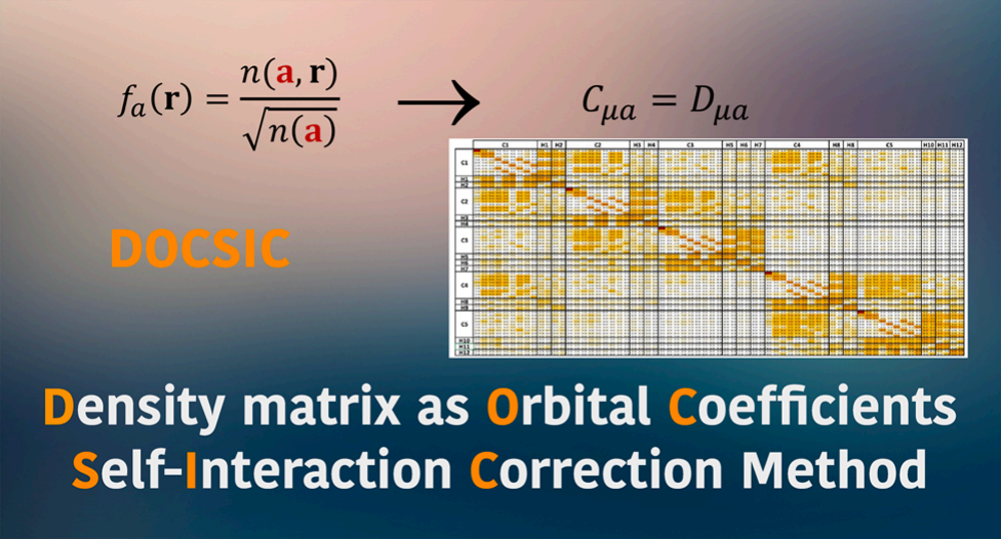

We introduce a new method to remove the one-electron self-interaction error in approximate density functional calculations on an orbital-by-orbital basis, as originally proposed by Perdew and Zunger [*Phys. Rev. B***1981**, *23*, 5048]. This method is motivated by a recent proposal by Pederson et al. [*J. Chem. Phys.***2014**, *140*, 121103] to remove self-interaction that employs orbitals derived from the real-space density matrix, known as FLOSIC (Fermi Löwdin orbitals self-interaction correction). However, instead of Fermi Löwdin orbitals, our scheme utilizes columns of the density matrix to determine localized orbitals, like the localization procedure proposed by Fuemmeler et al. [*J. Chem. Theory Comput.***2023**, *19*, 8572]. The new method, dubbed DOCSIC for density matrix as orbital coefficients self-interaction correction, contrasts with traditional Perdew–Zunger or FLOSIC in that it does not incorporate additional optimization parameters, and, unlike the average density self-interaction correction of Ciofini et al. [*Chem. Phys. Lett.***2003**, *380*, 12], it makes use of localized orbitals. Another advantage of DOCSIC is that it can be implemented as a mean-field formalism. We show details of the self-consistent generalized Kohn–Sham implementation, some illustrative results, and we finally highlight its advantages and limitations.

## 1 Introduction

Density functional theory (DFT)^1,2^ is widely used as the workhorse of electronic structure calculations thanks, in part, to its compromise between accuracy and computational efficiency.^3,4^ One of the most remarkable drawbacks of approximate exchange-correlation (XC) density functionals is their inability to cancel the self-interaction (SI) of electrons, which gives rise to the well-known self-interaction error (SIE) problem. SIE is the origin of several well-documented failures of lower-rung density-functional approximations, such as the incorrect potential energy dissociation curve for asymmetric species^5,6^ or the too high highest-occupied-molecular-orbital energy levels of negatively charged fragments, making their charge too positive.^7^

In 1981, Perdew and Zunger proposed a scheme to remove the one-electron SIE^8^ on an orbital-by-orbital basis. This scheme, usually known as Perdew–Zunger (PZ) self-interaction correction (SIC) is based on a modified orbital-dependent energy functional,
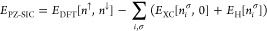
1where *n*_*i*_^σ^ are single orbital spin densities (σ = ↑, ↓) and *E*_XC_ and *E*_H_ are the exchange-correlation and Hartree energies, respectively. Minimizing the *E*_PZ-SIC_ functional leads to localized orbitals. In contrast to main-stream DFT, this scheme has not been adopted for routine applications due to the high computational cost associated with minimizing the energy, which involves finding a unitary transformation that minimizes *E*_PZ-SIC_. In addition, using the PZ-SIC scheme in combination with standard approximate functionals is harmful for many properties, and thus calls for the development of density functional approximations that are compatible with SIC, as highlighted in the pioneer work of Vydrov and Scuseria^9−11^ and, later, by others.^12−15^

Several approaches have been proposed to minimize *E*_PZ-SIC_. Some rely on the rigorous full relaxation of the (localized) orbitals utilizing a variety of techniques. All these methods differ in how they deal with the additional variational parameters introduced to represent localized orbitals, ranging from directly imposing localization equations conditions,^16−20^ to the constraint gradient search of Vydrov and Scuseria, to the unitary optimization-based two-step method of Lehtola and Jónsson,^21,22^ or to the work of Ferretti et al. with Koopmans-compliant functionals.^23^ Some implementations have been reported that utilize the optimized effective potential (OEP) technique in different flavors.^24−26^

Ciofini et al. proposed a simple approach, inspired by the original idea of Fermi and Amaldi, which consists of replacing the localized orbitals by a scaled fraction 1/*N* of the total density, with *N* being the number of electrons.^27^ The advantage of this so-called average-density SIC, or ADSIC, is that it leads to a mean-field set of equations that can be solved using standard self-consistent techniques and requires minimal additional computational cost. The disadvantage is that this method does not utilize localized one-electron orbitals. The scaled density can be seen as an ensemble density, and thus the XC functional in the PZ expression is not rigorously evaluated using one-electron densities.^28^ Both OEP and ADSIC approaches directly yield SI-corrected orbital energies, and thus can be used for evaluating ionization potentials and estimate excitation energies.

Another approach for minimizing *E*_PZ-SIC_ is based on the construction of Fermi-Löwdin orbitals.^29,30^ Within this approach, known as FLOSIC, the localized orbitals are parametrized in the form of Fermi orbitals,^31^
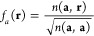
2where **a** are points in space, called Fermi orbital descriptors (FODs), and *n*(**r**′, **r**) is the density matrix (without considering spin indices, for clarity). This approach to construct localized orbitals was originally proposed by Luken et al.^32,33^ and can be seen as using the “rows” of a scaled density matrix as localized orbitals, taking advantage of the real-space decay of the density matrix for systems with a finite energy gap.^34,35^ The nonorthogonal Fermi orbitals *f*_*a*_(**r**) are orthogonalized using the Löwdin orthogonalization scheme^36^ to give place to the Fermi-Löwdin localized orthonormal orbitals (FLOs). The  denominator in eq 2 ensures that the orbitals have comparable weights during the Löwdin symmetric orthogonalization process. Since FLOSIC introduces the FODs as additional variational parameters, a full relaxation of the density and the entire set of *N*_occ_ FODs is needed to minimize *E*_PZ-SIC_. The first implementation of the FLOSIC methodology is based on Jacobi (or Givens^37^)-type rotations to zero the overlap between the occupied and virtual orbitals at each self-consistent iteration,^38^ much like standard implementations of Foster-Boys,^39,40^ Edmiston-Ruedenberg,^41^ Pipek-Mezey,^42^ or other localization schemes with various target functions,^43,44^ at fixed FODs. Recently, some of us have proposed a mean-field scheme for relaxing the density in FLOSIC.^45^ Other implementations of FLOSIC based on unified Hamiltonian schemes and effective potentials have been reported.^46−50^ In all these implementations, FODs are relaxed in-between self-consistent loops at fixed density in a double-loop fashion.

In this work we introduce a new approach to PZ-SIC inspired by the FLOSIC idea, which leads to an effective mean-field, generalized Kohn–Sham-like Hamiltonian that includes self-interaction correction, explicitly makes use of localized orbitals for SIE removal, and avoids introducing additional parameters in the calculations.

## 2 Theory and Implementation

The FLOSIC approach uses Fermi orbitals, eq 2, to construct localized orbitals, which are subsequently symmetrically normalized to build the Fermi-Löwdin orbitals, and, in turn utilized in the PZ scheme to remove SIE in an orbital-by-orbital basis. Fermi orbitals can be seen as localized orbitals, due to the localized nature of the density matrix in real space for finite systems.^34,35^ In analogy to FLOSIC, in this work we propose to utilize the density matrix in a localized, atom-centered representation, to construct localized orbitals. With this idea in mind, the coefficients of these localized orbitals can be simply extracted from single columns (or rows) of the atomic orbital density matrix. Since the elements of this matrix decay exponentially along a single row or column for finite systems (or extended systems with a finite bandgap), the resulting coefficients lead to localized orbitals. We note here that the normalizing denominator that appears in eq 2 is not needed as in the case of Fermi orbitals since, unlike the real-space density matrix, the atomic-orbital density matrix elements do not span orders of magnitude. This choice simplifies the algebra without affecting the results. We also note that this strategy is the same as one of the alternatives proposed by Damle and co-workers, called “selected columns of the density matrix”, or SCDM, in the context of providing noniterative localization schemes.^51,52^

In the FLOSIC scheme, the FODs, **a**, are additional variational parameters introduced to characterize the localized orbitals and, as such, they require to be included in the energy minimization procedure. In the currently proposed scheme, the role of the FODs is replaced by the choice of the column (or row) of the density matrix. Following the work on SCDM,^51,52^ we choose the columns using a rank-revealing QR factorization on a symmetrically orthonormalized density matrix. The procedure can be summarized as follows.

- First, we transform the density matrix in the nonorthonormal atomic orbital basis, **P**, to the Löwdin basis, **D** = **S**^1/2^**PS**^1/2^, where **S** is the overlap matrix in the atomic orbital basis. This symmetric orthonormalization ensures that the rank of the matrix is *N*_occ_ while keeping the matrix **D** as close to **P** as possible in the least-squares sense.^53^
- Next, a rank-revealing QR factorization is performed on **D**. The QR factorization with pivoting provides a solution to **DΠ** = **QR**, where **Q** and **R** are orthonormal and upper-triangular matrices, respectively, and **Π** is a permutation matrix, which in practice is a list of column indexes. The pivoting vector **Π** is chosen so that the diagonal elements of **R** are nonincreasing, i.e., |*R*_*ii*_| ≥ |*R*_*i*+1*i*+1_|. The first *N*_occ_ entries of **Π** (referred to as **π**) are then used to determine the coefficients **C** (see below). We note that only the first *N*_occ_ pivoting vector elements provided by the QR factorization are needed for this procedure, which, in practice, replaces the search for FODs in the FLOSIC scheme.
- Furnished with the **π** columns of the orthogonalized density matrix as coefficients, **C** = **D**_π_, the next step is to orthogonalize them. This can be done by first constructing the overlap matrix **O** = **C**^†^**C**, and then the orthogonalized coefficients are **X** = **S**^–1/2^**CO**^–1/2^.
- The single-orbital density matrices can be simply evaluated from the colummns of **X** as **P**^*a*^ = *X*_*a*_^†^*X*_*a*_

Choosing the π columns of **D** as coefficients ensures that the overlap matrix **O** is as well-conditioned as possible (the condition number for inversion is the lowest) and, thus, it is invertible. This, however, does not ensure that the SIC contribution to *E*_PZ-SIC_ (second term on the *r.h.s.* of eq 1) is minimal, but sets a protocol where the localized orbitals are uniquely defined from the atomic-orbital density matrix, which effectively avoids the incorporation of additional degrees of freedom in the SIC scheme.

A key capability of any approach that implements the PZ-SIC functional is to be able to minimize the PZ energy. In this case, the localization process does not introduce additional parameters or iterative procedures, and thus the resulting energy can be expressed solely in terms of the density matrix. This is a clear advantage since it implies that an effective multiplicative Kohn–Sham Hamiltonian can be defined, and the problem can be cast as a generalized Kohn–Sham scheme. To do this in practice, one can write the effective Kohn–Sham Hamiltonian as
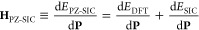
3where *E*_SIC_ refers to the second term on the *r.h.s* of eq 1,
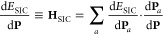
4In eq 3, d*E*_DFT_/d**P** ≡ **H**_DFT_ is the standard Kohn–Sham Hamiltonian, and in eq 4, d*E*_SIC_/d**P**_*a*_ = **H**_*a*_ is a single-orbital self-Hartree plus self-XC potential matrix. This proposed method is referred to as DOCSIC for density matrix as orbital coefficients self-interaction correction.

Table 1 summarizes some computational considerations for existing methods that implement the PZ scheme in different flavors. The ADSIC approach is the crudest but the most computationally effective. Traditional PZ, FLOSIC, and DOCSIC require the extra burden of evaluating additional XC and Hartree matrices. Depending on the particular implementation, FLOSIC may require additional XC and Hartree matrix evaluations during the Fermi-orbital descriptors relaxation. Table 1 illustrates the main advantage of the DOCSIC method, which is avoiding the incorporation of additional degrees of freedom while still using localized orbitals for SIE removal.

**Table 1:** Computational Cost Considerations for One-Electron SIE Removal Methods Based on PZ-SIC in the Generalized Kohn–Sham Scheme Compared to Standard DFT Calculations

	ADSIC	DOCSIC	FLOSIC	PZ
XC matrices/cycle^a^	1	*N*_occ_	*N*_occ_	*N*_occ_
Hartree matrices/cycle^a^	0	*N*_occ_	*N*_occ_	*N*_occ_
variational parameters^a^	0	0	3*N*_occ_	*N*_occ_(*N*_occ_ – 1)
localized orbitals?	no	yes	yes	yes
unitarily invariant?	n.a.	yes	yes	yes^b^

a In addition to standard DFT calculations.

b Imposed numerically in some cases.

We have implemented the DOCSIC approach using an in-house modification of the PySCF electronic structure code.^54^ The derivatives in eq 4 were evaluated using the automatic differentiation autograd from torch. Automatic differentiation techniques were introduced in quantum chemistry by Zhang and co-workers.^55^ Alternatively, one could follow the procedure outlined in ref (45) by some of us for the FLOSIC method. Most of the algebraic operations needed for the autograd derivative are readily available in the package torch, except for the forward and backward propagation of the matrix square root operation, which was added for this work. We note that, provided that the density matrix is real, the coefficients (and thus the localized orbitals) are real. The current implementation is restricted to real density matrices and hence to real-valued localized orbitals. After the generalized multiplicative Hamiltonian matrix of eq 3 is evaluated, the self-consistent procedure proceeds as any standard DFT calculation. The code is publicly available in https://github.com/peraltajuan/DOCSIC.

## 3 Test Calculations

There are several situations where SIE becomes the dominant source of errors in DFT calculations. For our first test calculations, we used the SIE4×4 test set of Grimme et al.^56^ for binding energies. This test set is a subset of the SIE11 set, originally devised to isolate SIE problems in thermochemistry. The SIE4×4 set focuses on “pure” one-electron SIE which are more difficult to capture in the SIE11 set.^56^ The test set consists of four positively charged dimers, H_2_^+^, He_2_^+^, (NH_3_)_2_^+^, and (H_2_O)_2_^+^ for which the dissociation energies at four points along their dissociation path were accurately calculated. These four points are at their respective intermonomeric equilibrium distance and 1.25, 1.5, and 1.75 times their equilibrium distance, and can be represented by a stretching factor Δ = 1.00, 1.25, 1.50, and 1.75, respectively. This test set was utilized in the past to assess the performance of different methods to correct energetic-based SIE.^57,58^ For these tests, as well as all other tests in this work, we use three representative density functional approximations from the first three rungs of Jacob’s ladder: LDA,^59−61^ PBE,^62,63^ and SCAN,^64^ in combination with the aug-cc-pVTZ basis set.^65,66^ For some calculations, we have used the maximum overlap method of Gilbert et al.^67^ to help self-consistent convergence. The errors for the functionals employed in this work for the SIE4×4 test set without SIE removal are typically large, with mean absolute deviations from reference data ranging from approximately 28 kcal/mol for LDA, 21 kcal/mol for PBE, and 18 kcal/mol for SCAN.^56,58^ As shown in Table 2, the DOCSIC method effectively removes one-electron self-interaction errors, as expected, bringing the dissociation energies much closer to the reference calculations than their respective uncorrected density functional counterparts.

**Table 2:** DOCSIC Dissociation Energies for the SIE4×4 set (in kcal/mol)^a^

	Δ	LSDA	PBE	SCAN	ref
He + He^+^ → He_2_^+^	1.00	63.1	52.6	56.6	56.9
	1.25	49.2	39.8	44.6	46.9
	1.50	31.3	22.4	27.6	31.3
	1.75	17.9	9.2	14.5	19.1
NH_3_ + NH_3_^+^ → (NH_3_)_2_^+^	1.00	39.0	29.8	29.5	35.9
	1.25	28.9	23.5	22.8	25.9
	1.50	16.1	12.2	11.4	13.4
	1.75	7.6	4.4	3.6	4.9
H_2_O + H_2_O^+^ → (H_2_O)_2_^+^	1.00	45.9	36.4	36.9	39.7
	1.25	33.5	28.3	28.3	29.1
	1.50	19.8	16.4	16.5	16.9
	1.75	11.7	9.8	10.0	9.3
RMSE		3.5	5.0	3.0	
ME		2.9	–3.7	–2.3	
RMSE^b^		3.1	4.4	2.6	
ME^b^		2.1	–2.8	–1.7	

a The one-electron H_2_^+^ case is excluded since PZ-SIC is exact. Coordinates and reference values are taken from ref (56). Δ represents the factor used to stretch the inter-atomic distances. The bottom rows include the root mean square and mean errors.

b Including the one-electron H + H^+^ → H_2_^+^ reactions.

It is worth recalling that in the traditional PZ approach, the matrix formed by elements ⟨*a*|**H**_*a*_|*b*⟩, with *a* and *b* occupied localized orbitals, is a Lagrange multiplier that is fully symmetric upon *E*_PZ-SIC_ being stationary, also known as Pederson’s localization equations.^16,68^ Moreover, the eigenvalues of the matrix constructed with elements λ_*ab*_ = ⟨*a*|**H**_DFT_ + **H**_*a*_|*b*⟩ can be interpreted as the ionization energies, since they are the derivative of *E*_PZ-SIC_ with respect to the occupation number.^16^ It should be emphasized that this Lagrange multiplier matrix does not play a role in the FLOSIC or DOCSIC methods, and can be constructed at a postprocessing stage. In the DOCSIC method, as well as in FLOSIC, the Lagrange multipliers **λ** matrix is not necessarily symmetric (but closely). This slight asymmetry is mathematically sound since the formalism does not impose any condition on the matrix, and it should be interpreted as the DOCSIC (and FLOSIC) solutions being slightly different than those obtained from the localization equations formalism. In this case, the ionization energies are thus approximated as the eigenvalues of the symmetrized **λ** matrix. Since ionization potentials are largely affected by one-electron self-interaction errors, we use this property as a second test to assess the capability of DOCSIC for SIE removal. In Table 3 we show representative results of ionization potentials for small hydrocarbons calculated using energy differences between the neutral and positively charged species (Δ*E*), from the highest occupied molecular orbital (HOMO) mean-field Kohn–Sham eigenvalue (−ε_HOMO_), and from the highest eigenvalue of the symmetrized **λ** matrix (−λ_HE_). As expected, the latest are much better approximations to Δ*E* than the mean-field eigenvalues.

**Table 3:** Ionization Potentials (in eV) for Hydrocarbons from Energy Differences (Δ*E*), HOMO Energies (ε_HOMO_) from Eigenvalues of the Generalized Kohn–Sham Hamiltonian, and of the Symmetrized Lagrange Multipliers Matrix (λ_HE_)

		Δ*E*	–ε_HOMO_	–λ_HE_
methane	LDA	13.64	9.37	13.49
	PBE	13.12	9.69	13.09
	SCAN	13.50	10.07	13.43
ethylene	LDA	10.41	6.64	10.40
	PBE	9.72	6.82	9.92
	SCAN	9.93	6.99	10.14
isobutene	LDA	9.06	5.96	9.58
	PBE	8.38	6.15	9.08
	SCAN	8.59	6.32	9.32

The appearance of broken-symmetry solutions in PZ-SIC was highlighted recently in the context of FLOSIC calculations by Hahn and co-workers.^69^ Without dwelling into the physical aspects of these solutions, we note that in DOCSIC, the SIC term added to the Kohn–Sham Hamiltonian, **H**_PZ-SIC_ (eq 3), is prone to introducing broken-symmetry solutions. We have explored the existence of broken-symmetry solutions by starting the calculations from several different initial guesses. In some cases, particularly in systems with high symmetry, two well-converged solutions that lie close in energy can be found. For example, for ethylene (*D*_2*h*_), we found a spin-compensated solution with a very small electric dipole moment of 0.145*D* (0.030 eÅ) along the C=C bond that lies 13 meV higher in energy than a slightly spin-polarized solution with ⟨*S*^2^⟩ = 0.008ℏ^2^ and zero electric dipole moment. None of the two solutions have the expected proper symmetry (either spin or space point-group), due to the inclusion of **H**_PZ-SIC_ in the Kohn–Sham Hamiltonian, which favors a spontaneous symmetry breaking. We also note that this characteristic could lead to convergence issues.

Another feature of the DOCSIC method is that the specific choice of the orbital coefficients taken from the density matrix columns given by the pivoting of the QR decomposition does not necessarily give the coefficients of the orbitals that minimize *E*_PZ-SIC_, preventing the method from being truly variational. Instead, these coefficients grant a unique, well-defined choice for the localized orbitals from the density matrix alone. We have explored this characteristic numerically by selecting as orbital coefficients all possible column permutations for small molecules with the 6-31G basis to keep the number of permutation manageable. We evaluated *E*_PZ-SIC_ for all permutations where the lowest eigenvalue of the overlap matrix **O** was larger than 10^–5^. We found that, indeed, the pivoting vectors that give the lowest condition number for **O**, do not coincide with those that yield the lowest *E*_SIC_. Moreover, oftentimes, solutions with a large condition number may correspond to solutions with lower *E*_SIC_. This issue also relates to potential complications in the FLOSIC method, where a gradient-based FOD relaxation to minimize *E*_SIC_ may lead to lower energy solutions with a badly conditioned Fermi-orbital overlap matrix. Alternatives to the QR decomposition with pivoting to determine localized orbitals will be explored in future work. Importantly, the results presented here highlight the potential of this method as a simple and effective way to remove SIE within the PZ scheme. A more extensive study considering larger molecules and test sets is underway.

## 4 Summary

We have introduced a method to remove the one-electron self-interaction error in approximate density functional calculations on an orbital-by-orbital basis, based on the PZ orbital-dependent energy functional. The method, referred to as density matrix as orbital coefficients self-interaction correction, or DOCSIC, utilizes columns of the atomic orbital density matrix to determine localized orbitals, like the localization procedure proposed by Fuemmeler et al.^51,52^ In contrast to full-fledged PZ or FLOSIC, our method does not incorporate additional variational parameters, and unlike the average density self-interaction correction of Ciofini et al.,^27,70^ our method explicitly utilizes localized orbitals for the PZ correction. We show details of the self-consistent generalized Kohn–Sham implementation and make the code publicly available. We demonstrate the applicability of DOCSIC with some illustrative results for the SIE4×4 test set and the ionization potentials of selected hydrocarbons. One of the main advantages of the method is that it can introduce localized orbitals without introducing additional parameters, while one of the drawbacks is that the localized orbitals are not those that necessarily minimize the PZ energy. However, the examples shown in this work demonstrate the capability of DOCSIC to correct for one-electron self-interaction errors in cases where these errors are dominant.
